# A Dataset of Lower Band Whistler Mode Chorus and Exohiss with Instrumental Noise Thresholds

**DOI:** 10.1038/s41597-025-05531-6

**Published:** 2025-07-18

**Authors:** Ondřej Santolík, Ivana Kolmašová, Ulrich Taubenschuss, Miroslav Hanzelka, David P. Hartley

**Affiliations:** 1 Department of Space Physics, Institute of Atmospheric Physics of the Czech Academy of Sciences, Prague, Czechia; 2 Faculty of Mathematics and Physics, Charles University, Prague, Czechia; 3 Department of Physics and Astronomy, University of Iowa, Iowa City, IA USA; 4 Present Address: Deutsches GeoForschungsZentrum, Helmholtz Centre, Potsdam, Germany

**Keywords:** Magnetospheric physics, Astrophysical plasmas

## Abstract

We describe a large database of natural electromagnetic emissions of lower band whistler mode chorus and exohiss within the Earth’s magnetosphere. It is based on more than 124 million selected survey measurements of magnetic fluctuations, recorded between 2001 and 2020 by the two NASA Van Allen Probes and four ESA Cluster spacecraft. The database provides a comprehensive view of amplitudes of these important electromagnetic emissions in the audible frequency range. We carefully condition the data to minimize the influence of instrumental artefacts. We also remove all data points which may be contaminated by instrumental noise using a newly developed method to define detection thresholds as a function of frequency, time, and instrument settings. The database can serve as a valuable resource for a broad range of scientists studying space weather, magnetospheric physics, and radiation belt dynamics.

## Background & Summary

Electromagnetic emissions of chorus and exohiss are a class of whistler-mode waves that occur naturally in the low-density regions of the Earth’s magnetosphere^1,2^ in the audible frequency band. Significant effects of these waves were found in the outer Van Allen radiation belt^3^. Knowledge of their properties therefore became essential for attempts at operational forecasting of radiation in the near-Earth environment^4,5^ with important societal implications^6^. Whistler-mode waves can rapidly increase highly variable fluxes of relativistic electrons^7–10^ or remove them into the atmosphere^11–14^. Previously, models of wave amplitudes were constructed based on the datasets of THEMIS spacecraft^15,16^, on the subset of measurements of the Cluster spacecraft and on Van Allen Probes measurements^17,18^. Although the original spacecraft datasets are accessible, a database of frequency-integrated amplitudes with removed instrumental artefacts, which is necessary for derivation of the chorus and exohiss models, was not made publicly available.

**Fig. 1:**
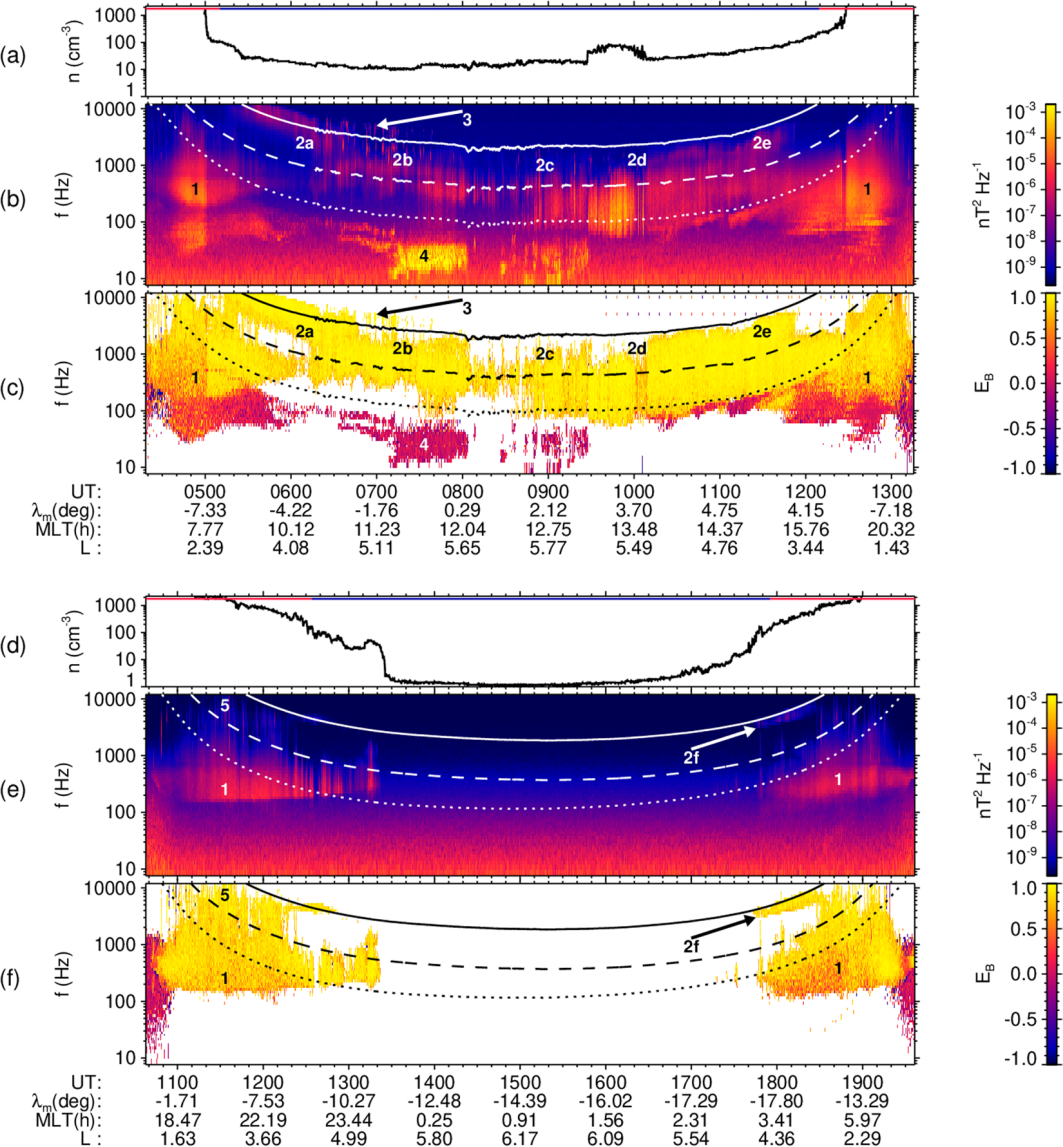
Examples of the source dataset. Survey data of the EMFISIS Waves instrument on Van Allen Probe A. The time interval corresponds to one orbital period of the spacecraft, from perigee to perigee. (**a**) Local plasma density from the upper hybrid frequency^21^ for a geomagnetically disturbed orbit of 28 September 2017; the red and blue horizontal line on the top indicates the position within the plasmapause and outside of it, respectively, according to an empirical model^20^. (**b**) Trace of the power spectral density matrix of the three magnetic field components as a function of frequency and time. (**c**) Signed magnetic field ellipticity^19^ plotted when the magnetic power spectral density is above the *P*_0_ = 10^−7^ noise threshold. (**d**–**f**) Plasma density, trace of the magnetic power spectral density matrix, and signed ellipticity for a geomagnetically calm orbit of 1 January 2015. Four types of whistler mode emissions are shown by numbers and arrows: 1—plasmaspheric hiss, 2a–f—lower band chorus and exohiss, 3—upper band chorus, 4—equatorial noise, 5—lightning generated whistlers. Overplotted dashed and solid lines show the frequency interval of lower-band exohiss/chorus between 0.1 and 0.5 ${f}_{{ce}0}$, where the equatorial electron cyclotron frequency ${f}_{{ce}0}$ is estimated from the locally measured magnetic field strength using Eq. 2. Dotted lines show high density estimates of the local lower hybrid frequency in a proton-electron plasma. Coordinates of the spacecraft are given at the bottom of each ellipticity plot: time UTC, magnetic latitude (${\lambda }_{m}$) in degrees, magnetic local time (MLT) in hours, and the *L* parameter in the dipole approximation.

Two examples of survey data acquired by the EMFISIS Waves instrument on Van Allen Probe A are shown in Fig. 1. We use the 3D measurement of the wave magnetic field to derive the trace of the magnetic power-spectral density matrix and estimate thus the power-spectral density of the squared modulus of the vector of magnetic field fluctuations as a function of frequency and time. We also use the same 3D measurement to obtain the magnetic ellipticity *E*_B_ in the polarization plane^19^ as a function of frequency and time. These characteristics, together with the position of the model plasmapause^20^, allow us to systematically distinguish different types of natural electromagnetic emissions by their frequency, characteristic polarization, and region of occurrence. For comparison, we also show the local plasma density determined from the measurements of the upper hybrid frequency^21^.

Figure 1a–c show measurements acquired after the geomagnetically disturbed period on 28 September 2017, when the Kp index was between 5- and 7-, and the model plasmapause position^20^ *L*_PP_ was between 2.8 and 3.3, roughly consistent with the measured transitions to the lower density regions along the spacecraft orbit. Intense whistler-mode waves were observed on the dayside. Figure 1d–f show measurements recorded after a period of calm geomagnetic conditions on 1 January 2015, when the Kp index was between 1 and 1+, and *L*_pp_ was between 4.5 and 4.6. No natural emissions of electromagnetic waves were observed between 14:00 and 17:00 UT on the nightside.

Plasmaspheric hiss^22^, shown as type 1 in Fig. 1b–c,e–f, typically occurs at characteristic frequencies between 20 Hz and 2 kHz, in the whistler mode with a right-handed polarization. It can be characterized by a significantly positive signed ellipticity^19^, *E*_B_ > 0.2. It is confined within the plasmasphere at *L* < *L*_PP_, where the *L* parameter is calculated from the spacecraft position using the dipole approximation,

1 $${\rm{L}}={\rm{R}}{\cos }^{-2}{\lambda }_{m},$$

where R is the radial distance from the Earth’s center in Earth radii (*R*_E_, defined as 6371.2 km), and ${\lambda }_{m}$ is the magnetic latitude related to the Earth’s main dipole axis.

This occurrence pattern is reversed for lower band chorus and exohiss^2,23^, which is the subject of this work and is shown as type 2a-2f in Fig. 1b,c,e–f. It occurs in the low-density plasmatrough at *L* > *L*_PP_, propagating in the whistler mode with *E*_B_ > 0.2. Its origin is linked to electron cyclotron resonance close to the geomagnetic equator^24–26^ and its typical frequency range between 0.1 ${f}_{{ce}0}$ and 0.5 ${f}_{{ce}0}$ is therefore related to the equatorial cyclotron frequency. Assuming propagation along the dipole magnetic field lines, we obtain it as

2 $${f}_{{ce}0}={f}_{{ce}}{\cos }^{6}{\lambda }_{m}/\sqrt{1+3\,{\sin }^{2}{\lambda }_{m}},$$

where ${f}_{{ce}}$ is the local electron cyclotron frequency obtained from measurement of the background magnetic field at the magnetic latitude ${\lambda }_{m}$.

Upper band chorus, shown as type 3 in Fig. 1b,c, occurs in the same region with similar polarization properties but at frequencies between 0.5 ${f}_{{ce}0}$ and 0.8 ${f}_{{ce}0}$. Equatorial noise^27,28^ is shown as an intense emission of type 4 in Fig. 1b,c. It occurs both inside and outside the plasmapause, at frequencies below the lower hybrid frequency (0.001 ${f}_{{ce}0}$ and 0.02 ${f}_{{ce}0}$) but with a polarization close to linear (|*E*_B_| < 0.2). Finally, lightning generated whistlers are observed as impulsive emissions in the plasmasphere (Type 5 in Fig. 1e,f).

**Fig. 2:**
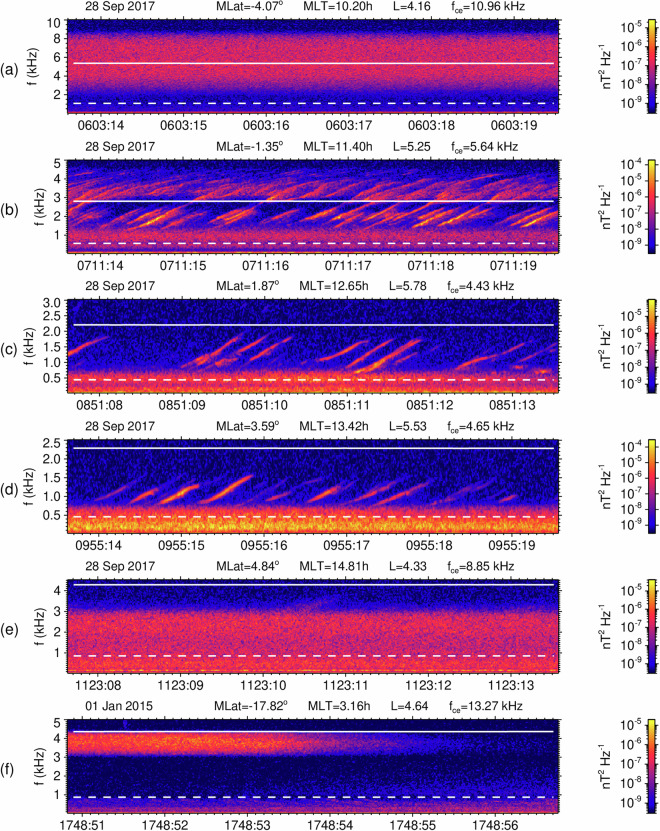
Examples of high-resolution spectrograms of lower-band chorus / exohiss. Panels a–f show the trace of the power spectral density matrix of the three magnetic field components as a function of frequency and time, obtained from 6 seconds long continuous burst mode intervals of the EMFISIS Waves instrument on Van Allen Probe A, for events marked 2a–f in Fig. 1. Spacecraft position and locally measured electron cyclotron frequency ${f}_{{ce}}$ are given on the top of each panel. Overplotted dashed and solid lines show the frequency interval of lower-band exohiss/chorus between 0.1 and 0.5 ${f}_{{ce}0}$, where the equatorial electron cyclotron frequency ${f}_{{ce}0}$ is estimated using Eq. 2. The waveforms, from which panels a–f have been obtained were transformed into stereo wave files (stored on 10.6084/m9.figshare.29433323.v1) using two magnetic field components perpendicular to the local magnetic field line.

These examples demonstrate that our dataset of three-dimensional measurements, together with the plasmapause model, allows us to distinguish chorus and exohiss from other types of magnetospheric wave emissions. However, it does not allow for distinguishing chorus from exohiss based on discrete time-frequency structures. This is only possible with high resolution measurements, an example of which are the continuous burst mode intervals of the EMFISIS Waves instruments onboard the Van Allen Probes. Figure 2 shows measurements from six such intervals, as they are marked 2a-2f in Fig. 1, corresponding to separate panels a-f in Fig. 2, respectively (hear also from audio files described below as Data record 13). The trace of the power spectral density matrix of the three magnetic field components as a function of frequency and time was calculated from high resolution 3D magnetic field measurements. The results show that either structureless exohiss or discrete chorus emissions or their various combinations can be observed in the frequency range between 0.1 ${f}_{{ce}0}$ and 0.5 ${f}_{{ce}0}$.

The high-resolution measurements shown in Fig. 2, however, are not continuously recorded but triggered from increased wave intensity. To avoid this selection effect, our procedure is based on the regularly sampled survey data, as they are described above and shown in Fig. 1. It therefore combines chorus and exohiss emissions, without distinguishing them based on their time-frequency structure. Each of the examples in Fig. 2 contains a 0.468 s long time interval of the survey mode capture at its very beginning. The frequency-integrated lower band chorus/exohiss amplitudes from these survey mode captures enter into our database. They are, in this case, approximately the same for both chorus and hiss, between 12 and 20 pT.

**Fig. 3:**
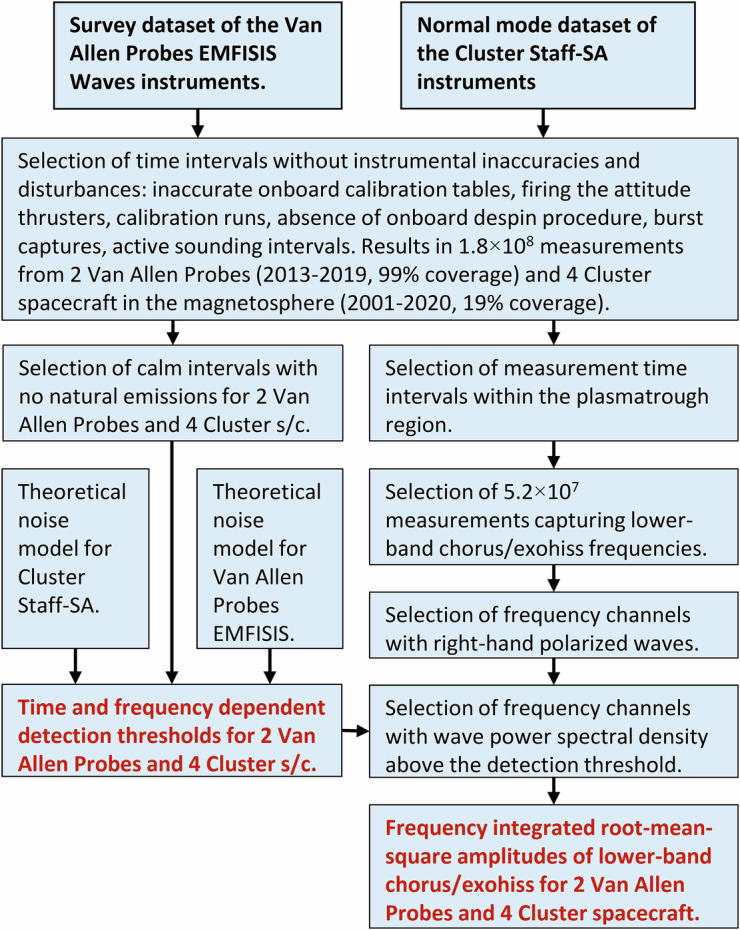
A scheme of the overall processing workflow to generate two categories of resulting Data records (in bold red).

Our analysis procedure is schematically depicted in Fig. 3. We collected a large dataset of observations from the systematic Survey mode measurements of the EMFISIS Waves instruments on the two NASA Van Allen Probes^29–31^ and from the Normal mode dataset of the STAFF-SA instruments on the four ESA Cluster spacecraft^32–34^ in the magnetosphere. From this combined dataset, we first removed all time intervals of known instrumental inaccuracies and disturbances, caused, for example, by inaccurate onboard calibration tables, firing the attitude thrusters, calibration runs, absence of onboard despin procedure, burst captures, or active sounding intervals.

This cleaned dataset first served for characterizing the detection thresholds by a manual selection of calm intervals containing only the instrumental noise, with no natural emissions of electromagnetic waves — such as the interval shown in the example Fig. 1e,f between 14:00 and 17:00 UT. We selected these intervals to cover the variations of the frequency dependent noise background for different measurements settings and to capture slow increases of the noise throughout the lifetime of the spacecraft caused by degradation of the instruments. The resulting frequency and time dependent detection thresholds are published as the first category of reusable Data records 1–6 described by the present paper.

We further used these detection thresholds to characterize the frequency integrated amplitudes of whistler-mode chorus and exohiss from the original cleaned data set — examples are shown as “type 2a–f” emissions in Fig. 1. We reduced the cleaned data set to time intervals of observations in the plasmatrough region, and to time intervals when the frequency interval of chorus/exohiss extended over at least one frequency channel of the instrument, while not extending outside its entire frequency range. We then integrated the power-spectral density of detected right-hand polarized waves over the frequency interval of chorus/exohiss, to obtain root-mean-square amplitudes. The database of these integrated amplitudes forms the second category of reusable Data records 7–12 described by the present paper.

The Methods section shows the details of the separate steps of a newly developed procedure to define detection thresholds and their evolution during the lifetime of the six spacecraft, and to obtain chorus/exohiss root-mean-square amplitudes. The Data Records section describes the files of the detection thresholds, which can be used generally throughout the original Van Allen Probes and Cluster datasets, not being limited to any preselected wave mode. This section also describes the files of resulting chorus/exohiss amplitudes derived from the measurements of the six spacecraft. The Technical Validation section demonstrates the validity of the plasmapause model and the consistency of the resulting data records, and, finally, the Usage Notes section shows examples and summarizes the usage of the new data records.

## Methods

### Survey dataset of the Van Allen Probes EMFISIS Waves instruments

The orbital coverage of two Van Allen Probes spacecraft allows us to systematically investigate the equatorial region for *L* < 6.7 at latitudes within 20° around the magnetic equator. The Level 2 Survey dataset of the EMFISIS Waves instrument^29–31^ can be obtained from NASA Space Physics Data Facility (on https://spdf.gsfc.nasa.gov/pub/data/rbsp/rbsp#/ l2/emfisis/wfr/spectral-matrix/, where ‘#’ is replaced by ‘a’ or ‘b’ for Van Allen Probe A or B, respectively).

The dataset consists of multicomponent spectral matrices obtained from measurements of three orthogonal magnetic search coil antennas and three electric double-probe antennas sampled at 35 kHz. The data were analyzed onboard the spacecraft using the fast Fourier transform of 16,384 samples with the von Hann’s cos^2^ windowing function in the time domain, based on 0.468 s long measurements repeated every 6 s. Resulting spectral matrices were arithmetically averaged into 65 frequency channels with a pseudo-logarithmic frequency spacing between 2 Hz and 12 kHz. As the original spectral estimates from the fast Fourier transform were linearly spaced in frequency, the number of averaged spectral matrices increased from *n*_*l*_ = 1 in the lowest 13 frequency channels (*l* = 1…13) up to *n*_65_ = 642 in the last frequency channel in order to achieve their pseudo-logarithmic spacing^29,30^.

Inaccurate onboard calibration tables were used by operational mistake on Probe A and Probe B before 12 and 13 February 2013, respectively, and we have removed the data acquired from the beginning of the mission up to these dates. The dataset from Van Allen Probe A therefore spans between 12 February 2013 and 13 October 2019, containing 3.48 × 10^7^ measurements with a 99.2% time coverage. The Van Allen Probe B dataset was recorded between 13 February 2013 and 16 July 2019 and contains 3.36 × 10^7^ captures with a 99.6% time coverage. All time intervals of known instrumental disturbances caused by firing the attitude thrusters have been subsequently removed from the datasets of both spacecraft, and the resulting dataset contains 3.45 × 10^7^ and 3.34 × 10^7^ measurements with 98.5% and 98.9% time coverage for Probe A and Probe B, respectively.

### Lower band chorus/exohiss dataset from the Van Allen Probes EMFISIS Waves instruments

We further selected only the measurements, for which the lower band chorus/exohiss frequency interval between 0.1 ${f}_{{ce}0}$ and 0.5 ${f}_{{ce}0}$ (see Eq. 2) was fully contained in the instrument frequency range, and at least one instrument frequency channel was entirely inside this interval. We additionally restricted our dataset to measurements inside the model magnetopause^35^, parametrized by the solar wind dynamic pressure and interplanetary magnetic field from the OMNI2 database^36^ (https://spdf.gsfc.nasa.gov/pub/data/omni/). All these conditions were fulfilled for 2.58 × 10^7^ and 2.51 × 10^7^ measurements of Van Allen Probe A and B, respectively. During that time, both probes had on average 14 frequency channels of the instrument entirely contained within the chorus/exohiss frequency interval.

### Normal mode dataset of the Cluster Staff-SA instruments

Four spacecraft of the Cluster mission have an eccentric high inclination orbit allowing us to investigate a wide range of latitudes up to at least 60° around the equator for *L* > 4. The Normal mode dataset of the STAFF-SA instrument^32–34^ can obtained from the European Space Agency Cluster Science Archive (using different retireval methods, for example through http://csa.esac.esa.int/csa-sl-tap/data?RETRIEVAL_TYPE=product&DATASET_ID=C#_CP_STA_ SM &START_DATE = 2004-07-15T00:00:00Z&END_DATE = 2004-07-16T00:00:00Z&DELIVERY_ FORMAT = CDF, where the START_DATE and the END_DATE can be set throughout the mission duration and where ‘#’ is replaced by 1, 2, 3, or 4, for Cluster 1, 2, 3, or 4, respectively).

The dataset contains spectral matrices obtained by onboard analysis of measurements by three orthogonal magnetic search coil antennas and two electric double-probe antennas, with measurements of 3.84 s repeated every 4 s and analyzed by numerical filtering in 27 logarithmically spaced frequency channels between 8 Hz and 4 kHz. They are divided into three separate groups of 9 channels, where the number of averaged spectral estimates is ${n}_{l}=4$, 32, and 256, in frequency channels $l=1\ldots 9$, 10…18, and 19…27, respectively.

The dataset was obtained by the four Cluster spacecraft between 7 January 2001 and 30 April 2020, resulting in 5.6 × 10^8^ measurements with 84%-time coverage. By excluding the measurements outside of the magnetosphere and in the distant tail at radial distances larger than 11 R_E_ we reduced the dataset down to 1.5 × 10^8^ measurements, corresponding to 22% time coverage. We subsequently removed all Burst mode intervals, intervals of active soundings of the Whisper^37^ instrument, calibration intervals, and intervals when the onboard de-spin procedure was switched off. The resulting cleaned dataset then contains 1.14 × 10^8^ measurements from all four Cluster spacecraft (2.82 × 10^7^, 2.88 × 10^7^, 2.80 × 10^7^, and 2.91 × 10^7^, from Cluster 1–4, respectively) corresponding to 19%-time coverage.

### Lower band chorus/exohiss dataset from the Cluster Staff-SA instruments

The dataset from the four Cluster spacecraft was further reduced to measurements inside the model magnetopause^35^, while measuring with at least one instrument channel fully inside the lower band chorus/exohiss frequency interval between 0.1 ${f}_{{ce}0}$ and 0.5 ${f}_{{ce}0}$ according to Eq. 2, and, at the same time, with this interval fully contained in the instrument frequency range. This yielded 1.84 × 10^7^, 1.77 × 10^7^, 1.80 × 10^7^, and 1.89 × 10^7^ measurements from Cluster 1–4, respectively, with an average number of 7 frequency channels of the instrument entirely contained within the chorus/exohiss frequency interval.

### Model of the cumulative distribution function for the noise power spectral densities of the magnetic field fluctuations for the dataset of the Cluster Staff-SA instruments

For the determination of the detection threshold we first need to determine the shape of the cumulative probability distribution ${F}_{l}\left({S}_{l}\right)$ of the trace ${S}_{l}$ of noise power spectral density matrix in each frequency channel $L$. Assuming normally distributed white noise with zero mean value for each of the sensors, the onboard spectral analysis results in power spectral densities obtained as the sum of squares of normally distributed real and imaginary parts of separate spectral components, which should therefore obey a scaled ${\chi }^{2}$ distribution with 2 degrees of freedom. These spectral estimates are further averaged across ${n}_{l}$ frequency bins and/or time intervals. The sum of power spectral densities from ${n}_{a}$ sensors (in our case *n*_*a*_ = 3) finally provides us with the trace of the ${n}_{a}$-dimensional power spectral density matrix. For independent spectral estimates we therefore theoretically obtain,

3 $${F}_{l}({S}_{l})={F}_{\nu }({\chi }^{2}),\nu =2{n}_{l}\,{n}_{a},\,{\chi }^{2}=\frac{{S}_{l}}{s},\,s=\frac{{\sigma }_{l}^{2}}{2\,{n}_{l}},$$

where ${F}_{\nu }$ is the cumulative probability of the *χ*^2^ distribution with $\nu$ degrees of freedom, and $s$ is a scaling factor, which depends on the original root-mean-square noise level ${\sigma }_{l}^{2}$ of the sensors in the given frequency channel $l$.

**Fig. 4:**
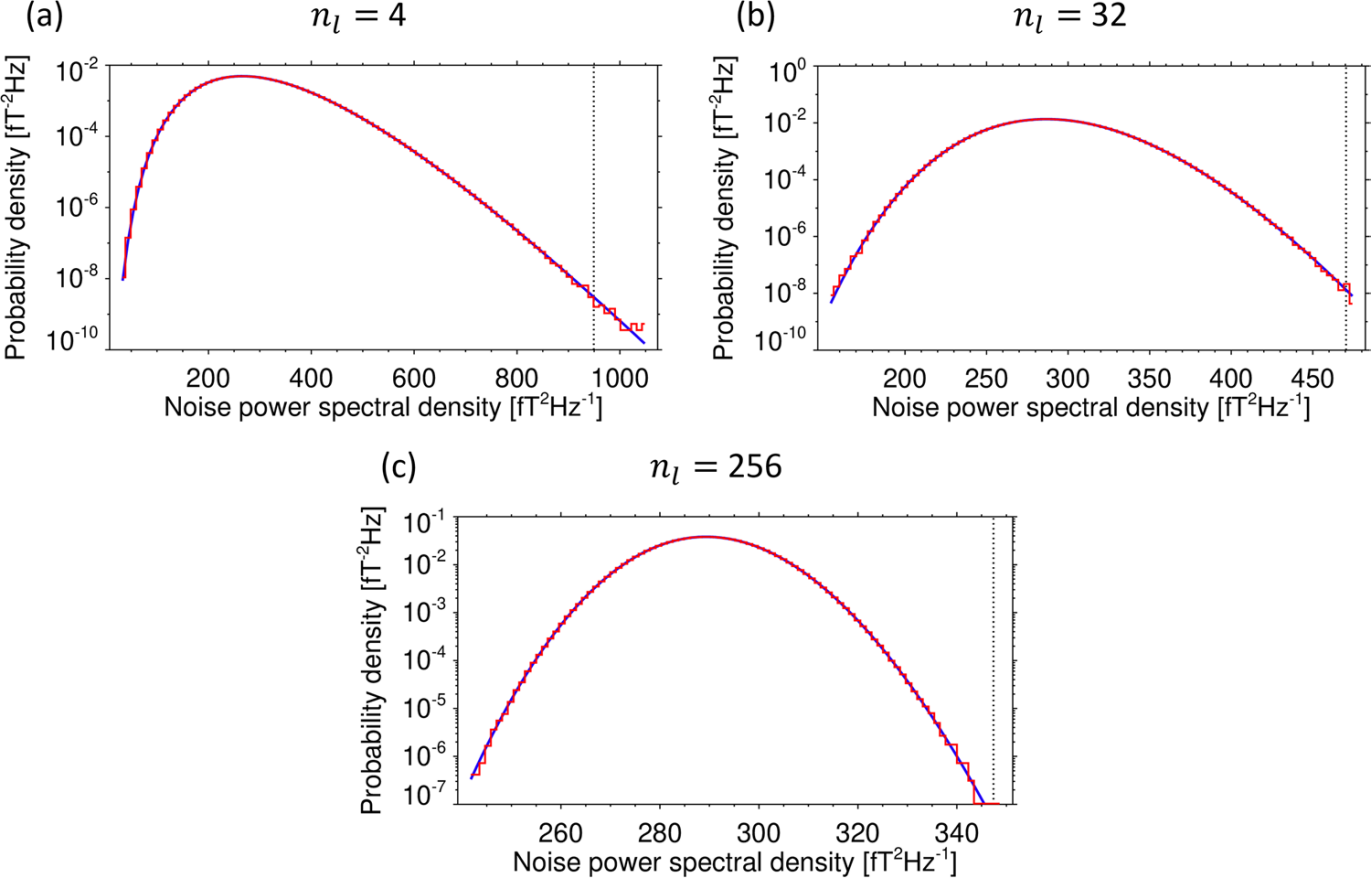
Probability density function of the simulated trace of the noise power spectral density matrix. (**a**) for *n*_*l*_ = $4$ averaged independent spectral estimates; (**b**) for *n*_*l*_ = 32; (**c**) for *n*_*l*_ = 256, relevant for the three groups of frequency channels of the Cluster STAFF-SA instruments. Red line: probability density estimated by a histogram from a Monte Carlo simulation of the sensor noise. Blue line: scaled ${\chi }^{2}$ distribution model according to Eq. 3. Vertical black dotted line: detection threshold ${S}_{0}$ (Eq. 6) for the trace of the power spectral density matrix to reach above ${S}_{0}$ with a probability *P*_0_ = 10^−7^.

We used a Monte Carlo simulation to verify this model by simulating the sensor noise with a normally distributed pseudo-random vector with a standard deviation $\sigma =$ 1.3 pT. This corresponds to the typical root-mean-square noise level of Cluster sensors integrated over their entire frequency band. We simulated the onboard spectral analysis using the Fast Fourier Transform (FFT) procedure over a pseudo-random noise sequence of 16384 samples, and we verified its normalization using the Parseval theorem by integrating the obtained power spectral densities over the entire frequency band, to retrieve the square of the original noise root-mean-square value $\sigma$. We then averaged power spectral densities in ${n}_{l}$ neighboring frequency bins. The procedure was repeated ${n}_{a}=$ 3 times and we summed the results to simulate the calculation of the trace of the magnetic power spectral density matrix. We constructed a histogram of obtained values from 2^18^ realizations of the pseudo-random noise sequences and normalized it to obtain estimates of the probability density function, which we compared with the first derivative of the cumulative probability distribution ${F}_{l}\left({S}_{l}\right)$ from Eq. 3. The results confirm a perfect agreement (Fig. 4), and we therefore used Eq. 3 to determine the detection thresholds for the Cluster STAFF-SA Normal mode data. With the measured noise intervals, we first determined the median value ${S}_{l,0.5}$ of the trace of the measured magnetic power spectral density matrices in each frequency channel $l$. With the appropriate number of averaged spectral estimates ${n}_{l}$ in this channel, we determined the original root-mean-square noise level ${\sigma }_{l}^{2}$ of the sensors from Eq. 3 by defining *F*_*l*_ (*S*_*l*,0.5_) = 0.5. We then used the obtained value of ${\sigma }_{l}^{2}$ in Eq. 3 together with Eq. 6 for determining the detection thresholds for the Cluster STAFF-SA Normal mode data.

### Model of the noise cumulative distribution function for power spectral densities of the magnetic field fluctuations for the Survey mode data products of the Van Allen Probes EMFISIS instruments

**Fig. 5:**
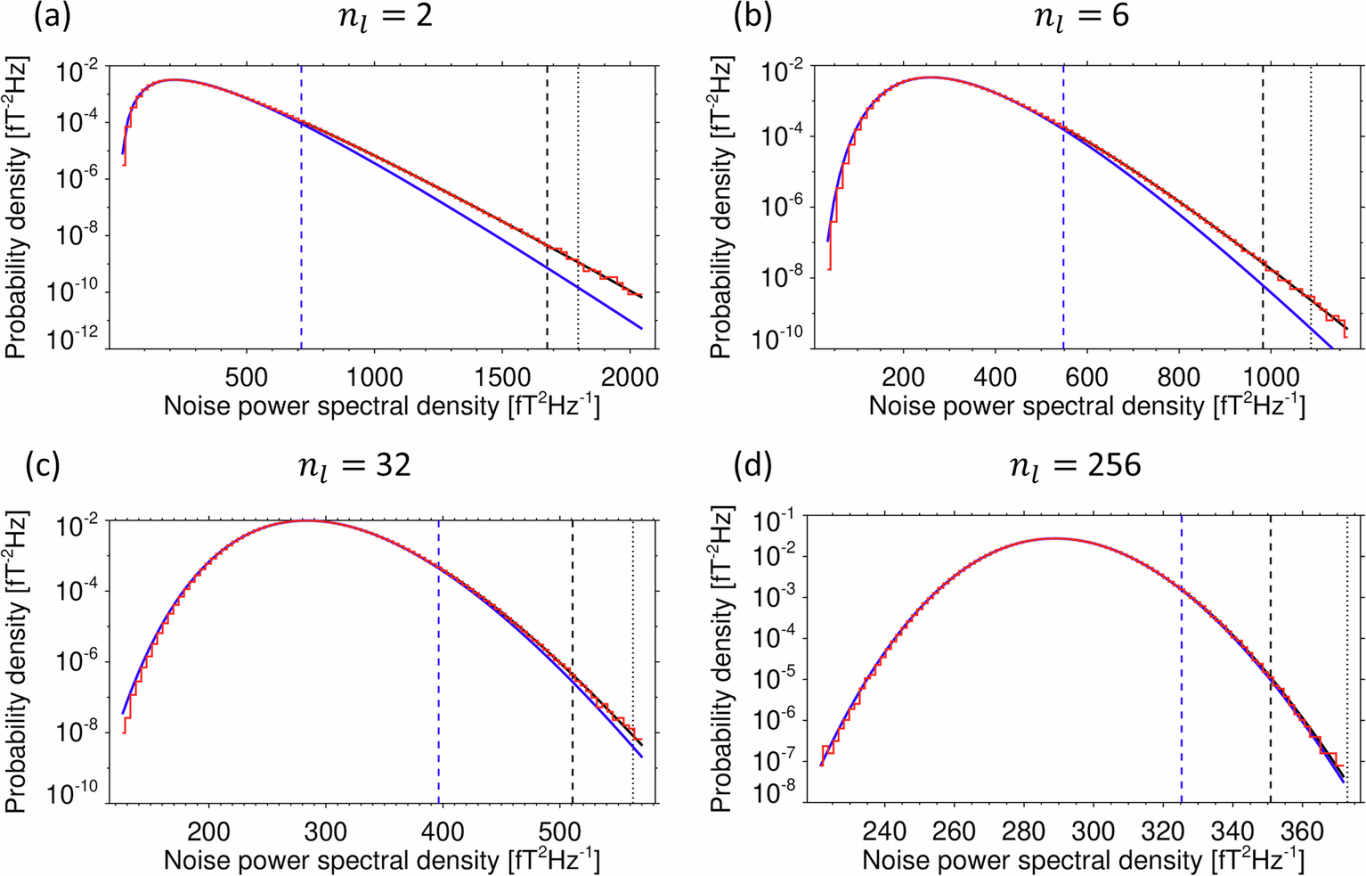
Probability density function of the simulated trace of the power spectral density matrix obtained from a windowed noise waveform. (**a**) for *n*_*l*_ = 2 averaged spectral estimates; (**b**) for *n*_*l*_ = 6; (**c**) for *n*_*l*_ = 32; (**c**) for *n*_*l*_ = 256, relevant for the Van Allen probes EMFISIS Survey data. Red line: probability density estimated by a histogram from a Monte Carlo simulation of the sensor noise. Blue line: scaled ${\chi }^{2}$ distribution core model from the first derivative of the cumulative probability distribution according to Eq. 4. The correction factors ${C}_{c\nu }$ and ${C}_{{cs}}$ for this model are determined by a nonlinear fit to the simulated histogram. The model is valid for noise power spectral densities, for which the cumulative distribution function is below 0.99, shown by the blue vertical dashed line. At larger power spectral densities, this core model significantly deviates from the simulation results. Black line: scaled ${\chi }^{2}$ distribution tail model according to Eq. 5, valid for noise power spectral densities, for which the cumulative distribution function is above 0.99. Black vertical dashed line: power spectral density for which the count in the simulated histogram decreases below 100, and random errors of the simulation start to be larger than 10%. The two vertical dashed lines also show the interval for the nonlinear fit of the correction factors ${C}_{t\nu }$ and ${C}_{{ts}}$ for the tail distribution model to the logarithm of the simulated histogram. Vertical black dotted line: detection threshold ${S}_{0}$ (Eq. 6) for the trace of the power spectral density matrix to reach above ${S}_{0}$ with a probability *P*_0_ = 10^−7^, according to the tail model from Eq. 5.

The onboard procedure of the Survey mode data products of the Van Allen Probes EMFISIS instruments included the von Hann’s cos^2^ windowing function for the measured time series to suppress the samples toward the edges of the analyzed time intervals. This violates the assumption of the statistical independence of the averaged neighboring spectral estimates, and the resulting probability distribution therefore deviates from the shape of the scaled ${\chi }^{2}$ distribution according to Eq. 3. To verify if this deviation is significant, we performed the same Monte Carlo simulation of the onboard procedure for this dataset as described above but we included the windowing function in the time domain. We also used a correction factor of 8/3 for power spectral densities obtained from the squared gain and noise bandwidth of the von Hann’s window^38^ to fulfill Parseval’s theorem. The results show that the distribution of the trace of the noise power spectral density matrix is significantly different from the model in Eq. 3 but that it can still be modeled by a combination of two separate scaled ${\chi }^{2}$ distributions ${F}_{{cl}}\left({S}_{l}\right)$ and ${F}_{{tl}}\left({S}_{l}\right)$ for the core and tail parts of the distribution, respectively (see Fig. 5). The core part is defined for ${S}_{l}$ such that *F*_*cl*_(*S*_*l*_) < 0.99,

4 $${F}_{{cl}}({S}_{l})={F}_{\nu }({\chi }^{2}),\nu =\frac{2\,{n}_{l}\,{n}_{a}}{{C}_{c\nu }},\,{\chi }^{2}=\frac{{S}_{l}}{s},\,s={\sigma }_{l}^{2}\frac{{C}_{{cs}}}{2\,{n}_{l}}.$$

The tail part is based on the same original root-mean-square noise level ${\sigma }_{l}^{2},$ but describes the high intensity part of the distribution occurring with a probability below 1%, it means for ${S}_{l}$ such that ${F}_{{cl}}\left({S}_{l}\right)$ ≥ 0.99,

5 $${F}_{{tl}}({S}_{l})={F}_{\nu }({\chi }^{2}),\nu =\frac{2\,{n}_{l}\,{n}_{a}}{{C}_{t\nu }},\,{\chi }^{2}=\frac{{S}_{l}}{s},\,s={\sigma }_{l}^{2}\frac{{C}_{{ts}}}{2\,{n}_{l}}.$$

**Fig. 6:**
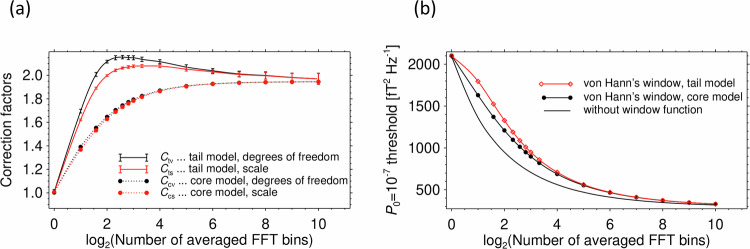
Correction factors and detection thresholds for the simulated trace of the power spectral density matrix obtained from a windowed noise waveform. (**a**) Correction factors from Eqs. 4 and 5, relevant to the Survey dataset of the EMFISIS Waves instrument on Van Allen Probes. Black dots: average values of the correction factor ${C}_{c\nu }$ for the number of degrees of freedom of the core distribution model; Red dots: average values of the correction factor ${C}_{{cs}}$ for the scale of the core distribution model; Black line with error bars: average values and standard deviations of the correction factor ${C}_{t\nu }$ for the number of degrees of freedom of the tail distribution model; Red line with error bars: average values and standard deviations of the correction factor ${C}_{{ts}}$ for the scale of the tail distribution model. The results are given as a function of the binary logarithm of the number ${n}_{l}$ of averaged spectral estimates; (**b**) Detection thresholds at *P*_0_ = 10^−7^, for the trace of the magnetic spectral density matrix in *n*_*a*_ = 3 dimensions, as a function of the binary logarithm of the number ${n}_{l}$ of averaged spectral estimates, assuming a constant sensor noise level of 100 fT^2^/Hz. Red diamonds: thresholds based on the tail distribution model from Eq. 5, which are used in our procedure, black dots: comparison with thresholds obtained from the core distribution model from Eq. 4, black solid line: comparison with thresholds based on the noise waveform without a window function, according to Eq. 3.

In Eq. 4, *C*_*cν*_, *C*_*cs*_ are, respectively, correction factors for the number of degrees of freedom and for the scale of the core part of the distribution. These factors effectively decrease the number ${n}_{l}$ of the averaged neighboring spectral estimates from Eq. 3, because these estimates are now correlated. Another set of correction factors, ${C}_{t\nu }$, ${C}_{{ts}}$ is used for the number of degrees of freedom and for the scale of the tail part of the distribution in Eq. 5. We determined these correction factors by numerical fits, based on the Monte Carlo simulation of the onboard procedure for this dataset. A set of 64 realizations of these fits (each of them based on 2^18^ realizations of the pseudo-random noise sequences of 16384 samples) resulted in average values and standard deviations of the four correction factors ${C}_{c\nu }$, ${C}_{{cs}}$, ${C}_{t\nu }$, and ${C}_{{ts}}$ for different ${n}_{l}$ values (Table 1 and Fig. 6). While the standard deviations of the corrections factors ${C}_{c\nu }$ and ${C}_{{cs}}$ for the core part of the distribution are small (below 0.1% in our simulation, not shown in Fig. 5), the corrections factors ${C}_{t\nu }$ and ${C}_{{ts}}$ for the tail part, reach larger standard deviations on the order of 2% but their average vales can still be used with a reasonable accuracy.

**Table 1:** Average values of correction factors from Eqs. 4 and 5. Results are obtained from Monte Carlo simulations for different values of the number of averaged spectral matrices ${n}_{l}$.

*n*_*l*_	*C*_*cν*_	*C*_*cs*_	*C*_*tν*_	${{\bf{C}}}_{{\boldsymbol{ts}}}$
1	1.003	1.006	1.009	1.005
2	1.391	1.368	1.695	1.621
3	1.553	1.532	2.007	1.89
4	1.646	1.629	2.116	1.998
5	1.704	1.689	2.149	2.043
6	1.744	1.731	2.155	2.062
7	1.772	1.761	2.151	2.071
8	1.794	1.784	2.149	2.077
10	1.824	1.816	2.136	2.079
16	1.87	1.865	2.116	2.078
32	1.908	1.906	2.07	2.052
64	1.927	1.926	2.039	2.03
128	1.937	1.936	2.012	2.007
256	1.942	1.941	1.999	1.996
512	1.944	1.944	1.982	1.981
1024	1.945	1.945	1.97	1.97

With the measured noise intervals, we first determined the median value ${S}_{l,0.5}$ of the trace of the measured magnetic power spectral density matrices in each frequency channel $l$. With correction factors ${C}_{c\nu }$, ${C}_{{cs}}$ for the appropriate number of averaged spectral estimates ${n}_{l}$ in this channel, we determined the original root-mean-square noise level ${\sigma }_{l}^{2}$ of the sensors from Eq. 4 for the core model median by defining ${F}_{{cl}}\left({S}_{l,0.5}\right)=$ 0.5. We then used the obtained value of ${\sigma }_{l}^{2}$ in Eq. 5 for the tail model and we used it together with Eq. 6 for determining the detection thresholds for the Survey mode data products of the Van Allen Probes EMFISIS instruments.

### Determination of detection thresholds

We have characterized the instrumental noise for each spacecraft separately by manual selection of a series of measurement intervals, in which the signal of natural wave emissions was absent (as it was the case for 14:00–17:00 UT on 1 January 2015, shown in Fig. 1d–f). We assumed models of the probability distribution of the trace of the power spectral density matrix for both the Survey dataset of the Van Allen Probes EMFISIS Waves instruments (Eqs. 4 and 5), and the Normal mode dataset of the Cluster STAFF-SA instruments (Eq. 3). The models are based on scaled ${\chi }^{2}$ distributions with the number of degrees of freedom defined by known properties of the onboard analysis in each frequency channel, and with a scaling factor, which depends on the noise level of the sensors, as detailed below. This is the only free parameter of the model, which we determined from a robust estimate of the median value of the trace of the power spectral density matrix in each of the analyzed noise intervals.

**Fig. 7:**
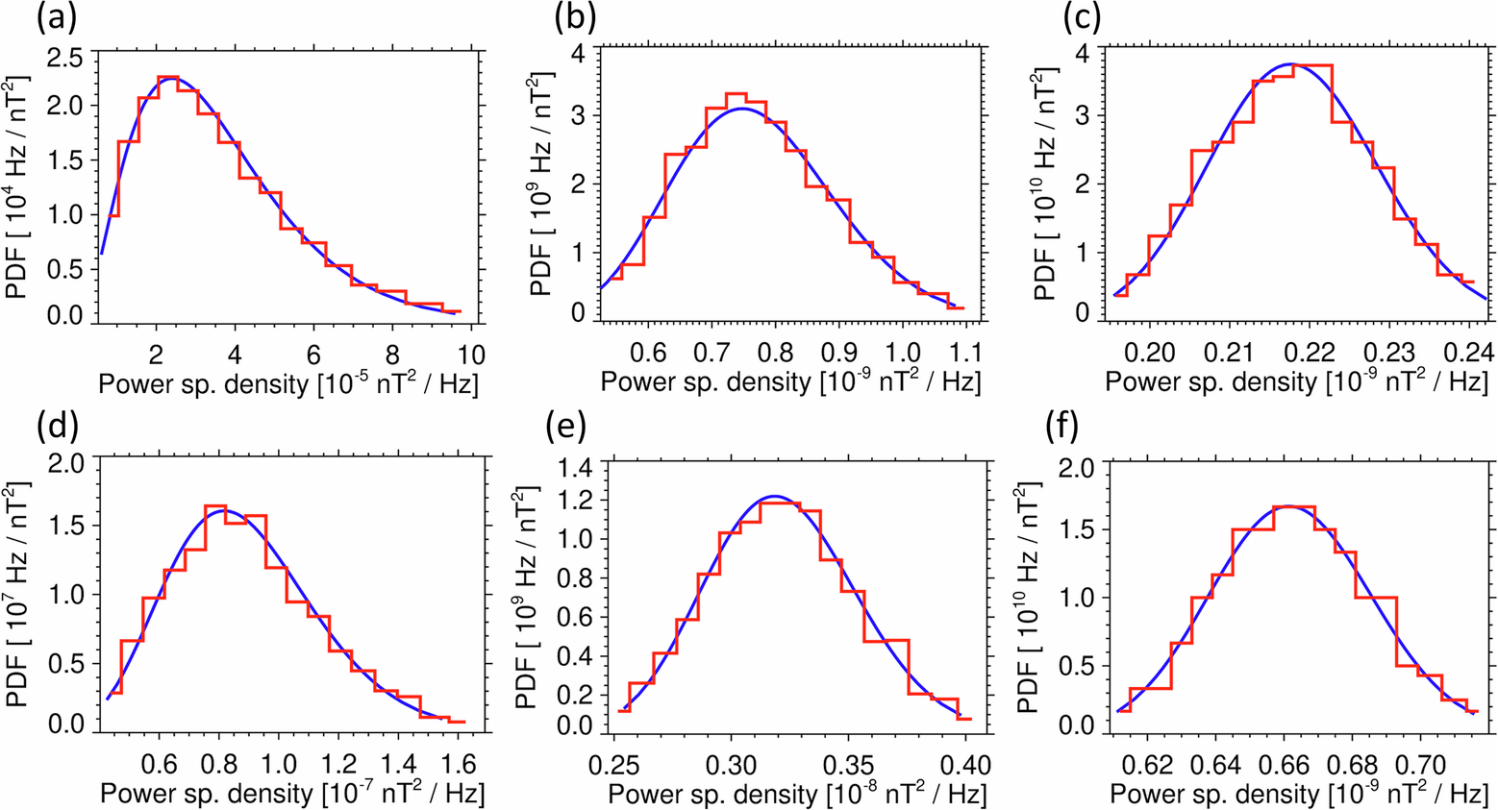
Probability density function (PDF) of the trace of the magnetic power spectral density matrix. Normalized histograms were obtained from the Survey data measured by the EMFISIS Waves instrument onboard Van Allen Probe A between 14:00 and 17:00 UT on January 1, 2015: (**a**) channel number^29^ *l* = 2, central frequency *f*_*l*_ = 4 Hz, number of averaged power spectra *n*_*l*_ = 1; (**b**) *l* = 36, *f*_*l*_ = 397 Hz, *n*_*l*_ = 22; (**c**) *l* = 58, *f*_*l*_ = 5011 Hz, *n*_*l*_ = 271, and from the STAFF-SA instrument onboard the Cluster 1 spacecraft between 18:00 and 21:00 UT on March 8, 2002: (**d**) the 6^th^ frequency channel with a central frequency of 28 Hz and *n*_*l*_ = 4 averaged spectra; (**e**) the 13^th^ channel at 141 Hz and with *n*_*l*_ = 32 averaged spectra; (**f**) the 24^th^ channel at 1794 Hz, with *n*_*l*_ = 256 averaged spectra. Blue curves show results of the models based on the scaled ${\chi }^{2}$ distributions^24^.

**Fig. 8:**
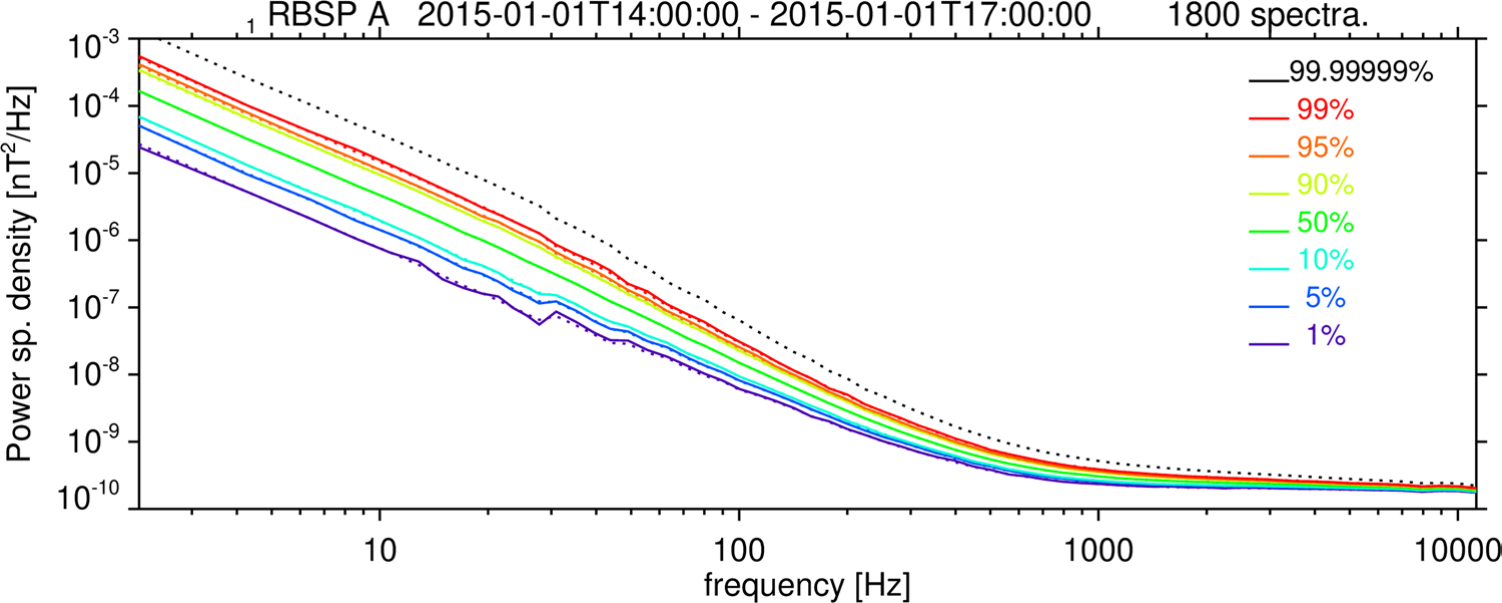
Distribution of Survey data search coil noise for the Van Allen Probe A EMFISIS instrument. Percentiles of the probability distribution of the trace of the magnetic power spectral density matrix as a function of frequency ${f}_{l}$, obtained from all 65 frequency channels $l$ of Survey data measured by the EMFISIS Waves instrument onboard Van Allen Probe A between 14:00 and 17:00 UT on January 1, 2015, and corresponding to examples from Fig. 2. The colored dotted lines show the theoretical cumulative probabilities of the core model distribution according to Eq. 4, with the root-mean-square noise level ${\sigma }_{l}^{2}$ determined using the observed median value. The black dotted line corresponds to the *P*_0_ = 10^−7^ detection threshold from the tail model distribution according to Eqs. 5 and 6, with the same ${\sigma }_{l}^{2}$.

**Fig. 9:**
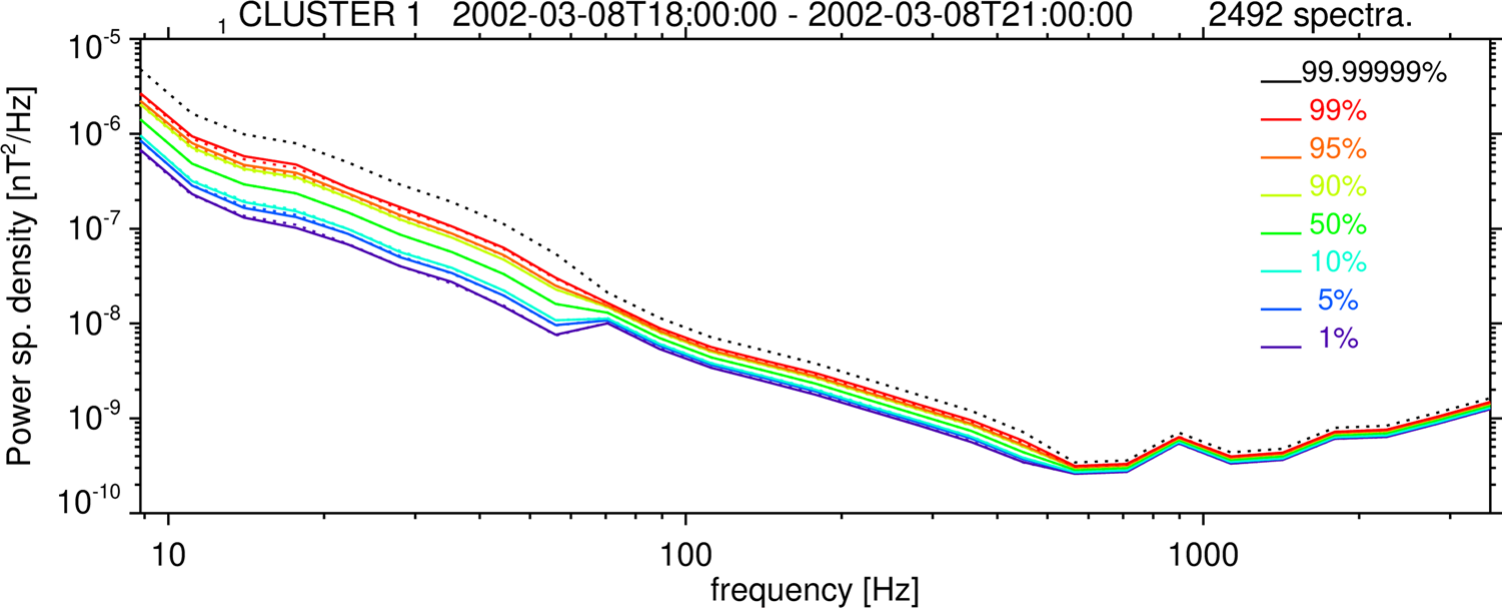
Distribution of search coil noise for Cluster 1 STAFF-SA instrument. Percentiles of the probability distribution of the trace of the magnetic power spectral density matrix of the instrumental noise as a function of frequency ${f}_{l}$ obtained from all 27 frequency channels $l$ of the STAFF-SA instrument onboard Cluster 1. The data were acquired during a calm interval on March 8, 2002 from 18:00 to 21:00 UT. Dotted lines show theoretical cumulative probabilities of the scaled ${\chi }^{2}$ distribution according to Eq. 3, with the root-mean-square noise level ${\sigma }_{l}^{2}$ determined using the observed median value, and with $\nu =2\,{n}_{l}\,{n}_{a}$ degrees of freedom, where *n*_*a*_ = 3 is the number of search coil antennas, and ${n}_{l}$ is the number of onboard averaged power spectra. *n*_*l*_ = 4 for *l* = 1…9, *n*_*l*_ = 32 for *l* = 10…18, *n*_*l*_ = 256 for *l* = 19…27. The black dotted line corresponds to *P*_0_ = 10^−7^ from Eqs. 3, 6.

The agreement of this model with experimental data is shown by examples in Fig. 7 using Van Allen probes EMFISIS Waves measurements during the noise interval on 1 January 2015 (Fig. 1d–f) and Cluster STAFF-SA measurements from a noise interval on 8 March 2002. The experimentally determined percentiles of the noise distribution between 1% and 99% also correspond well to the model across the entire frequency range of these instruments (see Figs. 8 and 9). We can therefore use this model to calculate the detection threshold ${S}_{0l}$ for the trace of the power spectral density matrix in a frequency channel $l$ such that the noise level can reach above ${S}_{0l}$ with an arbitrarily chosen low probability:

6 $${P}_{0}=1-{F}_{l}\left({S}_{0l}\right),$$

where ${f}_{l}$ is the modeled cumulative probability distribution (according to Eqs. 3–5).

The average probability that the instrumental noise can randomly overpass the power-spectral density threshold for at least one frequency channel of the instrument can be estimated as

7 $$P=1-{\sum }_{s}{N}_{s}{(1-{P}_{0})}^{{K}_{s}}/{\sum }_{s}{N}_{s},$$

where ${P}_{0}$ is the predefined probability for the determination of the detection threshold for each instrumental frequency channel, ${N}_{s}$ is the number of observations by a spacecraft $s$, and ${K}_{s}$ is the number of instrument channels inside the lower band chorus frequency interval averaged over the ${N}_{s}$ observations in our dataset of lower-band chorus/exohiss. Here, the total number of observations is *N*_1_ = 5.1 × 10^7^ for both Van Allen Probes, with *K*_1_ = 14, and is *N*_2_ = 7.3 × 10^7^ for all Cluster spacecraft, with *K*_2_ = 7 (see above in the dataset description).

If we limit ourselves to the highest experimentally determined percentiles of the noise distribution, we still have a large fraction of cases where instrumental noise causes random false positive results, considering that they can occur anywhere in the analyzed frequency band. Using a percentile of 99% (*P*_0_ = 0.01), we obtain a quite large probability $P$ of 9.4% of false positive detections according to Eq. 7, which would then substantially bias the determined occurrence rates. For practical purposes, we therefore must use the model distribution to extend the measurements to high cumulative probabilities (it means low ${P}_{0}$). To have a sufficiently low broadband probability of false detections, we define the cumulative probability threshold for each frequency channel to be *P*_0_ = 10^−7^. Following Eq. 7, this leads to the resulting probability *P* = 10^−6^, giving on average only 124 false positive detections out of the entire dataset of 1.24 × 10^8^ suitable measurements in the magnetospheric region and frequency range of chorus or exohiss.

Using *P*_0_ = 10^−7^ may seem too restrictive, but in fact, it does not substantially increase the noise threshold values compared to lower percentiles, especially at higher frequency channels where more spectral estimates are averaged onboard the spacecraft (Fig. 8). This is primarily where chorus/exohiss observations occur. Analysis of similar cases of noise intervals collected during the Van Allen Probes operational mission shows that, as the preamplifier electronics of the search coil sensors degraded during the mission, the noise level increased. We took this evolution into account in our analysis, and we also considered the setting of the signal attenuator which substantially influenced the detection threshold at higher frequencies. The results^39^ are stored as Data records 1 and 2.

We used the same time-dependent method to find the detection threshold of power spectral densities also for the measurements of the four Cluster spacecraft (see Fig. 9 and Data records 3–6)^39^.

### Root-mean-square amplitudes of lower band chorus/exohiss

We used these newly derived data records of detection thresholds together with the results of the selection procedure described by the first four paragraphs on the Methods section to define the database of root-mean-square amplitudes of the lower band chorus/exohiss. The data selection procedure yielded measurements from the Normal mode dataset of the Cluster Staff-SA instruments and from the Survey mode dataset of the Van Allen Probes EMFISIS Waves instruments, for which the lower-band of chorus/exohiss was fully contained in the instrument frequency range, and, at the same time, at least one instrument frequency channel was entirely contained inside this interval.

Each selected measurement was obtained in the form of multidimensional power-spectral matrices in one or more frequency channels of each instrument. From a 3D magnetic power-spectral matrix in a frequency channel $l$ we calculated the signed magnetic ellipticity ${E}_{{Bl}}$ in the polarization plane^19^. In the same frequency channel, we also calculated the trace ${S}_{{Bl}}$ of the magnetic power-spectral density matrix to estimate the power-spectral density of the squared modulus of the vector of magnetic field fluctuations. We then calculated the root-mean-square amplitude:

8 $${{\rm{B}}}_{{\rm{w}}}=\sqrt{{\sum }_{\{l:{E}_{{Bl}} > 0.2,{S}_{{Bl}} > {S}_{0l}\}}\,{S}_{{Bl}}\,{\varDelta }_{l}},$$

where ${\varDelta }_{l}$ is a frequency bandwidth of channel $l$, and where the sum is calculated over all frequency channels, which were entirely contained in the lower-band chorus/exohiss frequency interval, whose signed magnetic ellipticity overpassed a threshold of 0.2, and whose trace of the magnetic power-spectral density matrix overpassed the detection threshold ${S}_{0l}$. The resulting root-mean-square amplitudes are given as a function of time and position of each measurement in Data records 7–8 for Van Allen Probes A and B, respectively, and in Data records 9–12 for Cluster 1–4, respectively^39^.

## Data Records

**Data record 1: Detection thresholds for Survey data of the EMFISIS instrument on Van Allen Probe A**.

10.6084/m9.figshare.27606945

**Filename: RBSPA_SURV_BSUM_NOISE.txt**

The ASCII text file contains

- Self-explanatory comments introduced by a “%” sign, starting by a header of the file on its first line;
- human and machine readable numerical data.

To capture the time evolution of the noise floors, with a possibility of sudden steps linked to the changes in settings of the instrument, a sufficiently general format of the numerical data is used. First a list of time nodes between which the noise floors are interpolated is given, followed by the noise floors themselves. The data are therefore listed in two sections

- table of time nodes,
- table of noise floors.

- The table of time nodes starts on the second line of the file, where the number of lines in the table is given. The table then starts, after a self-explanatory line of comments, on the fourth line of the file, each of its lines contains the following three data components separated by spaces:

- Universal time in the “YYYY-MM-DDThh:mm:ss.msc” format, where “YYYY” stands for a 4-digit calendar year, “MM” for a 2-digit calendar month, “DD” for a 2-digit calendar day, “hh” for a 2-digit hour,”mm” for a 2-digit minute, “ss” for a for a 2-digit second, and “msc” for a for a 3-digit millisecond;
- noise floor number from the table of noise floors (starting by 0, see below) to which the noise floor should be time-interpolated between the previous time node and the current time node;
- noise floor number from the table of noise floors (starting by 0, see below) from which the noise floor should be time-interpolated between the current time node and the following time node.

Note that these two noise floor numbers may be identical to account for slow changes linked to degradation of the sensors, but also different, to account for the sudden steps linked to the changes of instrument settings.

b) The table of noise floors immediately follows the table of time nodes. It starts by a line defining the number of noise floors and number of frequency channels contained in each of them. Each noise floor is then described by two lines:

- a comment line defining the noise floor number (starting from 0), the spacecraft and time interval, over which the noise floor analysis was done, the number of noise spectra included in the analysis, and the probability threshold ${P}_{0}$;
- a sequence of numerical values for the noise floor in nT^2^/Hz. Each of these values is equal to the detection threshold ${S}_{0l}$ for the $l$ th frequency channel according to Eq. 6 with *P*_0_ = 10^−7^.

An example of using this data record for obtaining a noise floor at a given time is described in the Usage Notes section. A simple custom code to handle the tables of detection thresholds is available on 10.6084/m9.figshare.29433593.

**Data record 2: Detection thresholds for Survey data of the EMFISIS instrument on Van Allen Probe B**.

10.6084/m9.figshare.27606945

**Filename: RBSPB_SURV_BSUM_NOISE.txt**

The format is the same as for the Data record 1.

**Data record 3: Detection thresholds for Normal mode data of the STAFF-SA instrument on Cluster 1**.

10.6084/m9.figshare.27606945

**Filename: C1_STAFFSA_BSUM_NOISE.txt**

The format is the same as for the Data record 1.

**Data record 4: Detection thresholds for Normal mode data of the STAFF-SA instrument on Cluster 2**.

10.6084/m9.figshare.27606945

**Filename: C2_STAFFSA_BSUM_NOISE.txt**

The format is the same as for the Data record 1.

**Data record 5: Detection thresholds for Normal mode data of the STAFF-SA instrument on Cluster 3**.

10.6084/m9.figshare.27606945

**Filename: C3_STAFFSA_BSUM_NOISE.txt**

The format is the same as for the Data record 1.

**Data record 6: Detection thresholds for Normal mode data of the STAFF-SA instrument on Cluster 4**.

10.6084/m9.figshare.27606945

**Filename: C4_STAFFSA_BSUM_NOISE.txt**

The format is the same as for the Data record 1.

**Data record 7: Amplitudes of lower-band chorus and exohiss from Survey data of the EMFISIS instrument on Van Allen Probe A**.

10.6084/m9.figshare.27606945

**Filename: VA_LBall_Bw_2025Mar28_133831.txt**

The selection procedures for this data record are described in the first two paragraphs of the Methods section. The ASCII text file contains:

- a one-line header describing the contents of the following data items;
- a sequence of human and machine readable data items, each on a separate line, containing a list of 7 data components separated by spaces:

1. date of universal time UTC in the “YYYY-MM-DD” format, where “YYYY” stands for a 4-digit calendar year, “MM” for a 2-digit calendar month, “DD” for a 2-digit calendar day
2. time from last midnight of universal time UTC in the in the “hh:mm:ss” format, where “hh” stands for a 2-digit hour,”mm” for a 2-digit minute, “ss” for a for a 2-digit second;
3. Root-mean-square amplitude B_w_ of lower-band chorus and exohiss in pT from Eq. 8; zero means that the measured power-spectral densities were below the noise threshold in all frequency channels.
4. Magnetic local time MLT in hours derived from the solar magnetic (SM) coordinates^40^ of the spacecraft as the azimuth angle in the ${x}_{{SM}}$ – ${y}_{{SM}}$ plane;
5. Magnetic latitude ${\lambda }_{m}$ related to the Earth’s main dipole axis, given in degrees, and derived again from the solar magnetic (SM) coordinates of the spacecraft^40^ as the angle from the ${x}_{{SM}}$ – ${y}_{{SM}}$ plane;
6. Radial distance $R$ from the center of the Earth, given in Earth radii, and derived as the distance from the origin of the solar magnetic (SM) coordinates of the spacecraft^40^, using the definition of one Earth radius equal to 6371.2 km;
7. Difference $L-{L}_{{PP}}$ of the equatorial distance of a dipole field line passing through the spacecraft at the time of measurement (with an $L$ value from Eq. 1) from the model plasmapause ${L}_{{PP}}$ ^20^.

**Data record 8: Amplitudes of lower-band chorus and exohiss from Survey data of the EMFISIS instrument on Van Allen Probe B**.

10.6084/m9.figshare.27606945

**Filename: VB_LBall_Bw_2025Mar28_133831.txt**

The format is the same as for the Data record 7.

**Data record 9: Amplitudes of lower-band chorus and exohiss from Normal mode data of the STAFF-SA instrument on Cluster 1**.

10.6084/m9.figshare.27606945

**Filename: C1_LBall_Bw_2025Mar28_133831.txt**

The format is the same as for the Data record 7. The selection procedures for this data record are described in the third and fourth paragraphs of the Methods section.

**Data record 10: Amplitudes of lower-band chorus and exohiss from Normal mode data of the STAFF-SA instrument on Cluster 2**.

10.6084/m9.figshare.27606945

**Filename: C2_LBall_Bw_2025Mar28_133831.txt**

The format is the same as for the Data record 7. The selection procedures for this data record are described in the third and fourth paragraphs of the Methods section.

**Data record 11: Amplitudes of lower-band chorus and exohiss from Normal mode data of the STAFF-SA instrument on Cluster 3**.

10.6084/m9.figshare.27606945

**Filename: C3_LBall_Bw_2025Mar28_133831.txt**

The format is the same as for the Data record 7. The selection procedures for this data record are described in the third and fourth paragraphs of the Methods section.

**Data record 12: Amplitudes of lower-band chorus and exohiss from Normal mode data of the STAFF-SA instrument on Cluster 4**.

10.6084/m9.figshare.27606945

**Filename: C4_LBall_Bw_2025Mar28_133831.txt**

The format is the same as for the Data record 7. The selection procedures for this data record are described in the third and fourth paragraphs of the Methods section.

**Data record 13: Sound files for data in** Fig. 2**in the wav format**.

10.6084/m9.figshare.29433323

This figshare dataset collects examples of 6 stereo sound files in the wav format, 2a.wav, 2b.wav, 2c.wav, 2d.wav, 2e.wav, and 2f.wav, related to the corresponding panels a, b, c, d, e, and f in Fig. 2. Each file contains a sequence of 208896 measured samples of two components of the fluctuating magnetic field, which are perpendicular to the background magnetic field line. The first (left-side) stereo channel is used for the component in the plane of the local magnetic meridian, while the component perpendicular to this plane is stored in the second (right-side) channel. No additional processing is used, and the original sampling frequency of 35 kHz is conserved.

## Technical Validation

Each of the Data records 1–2, containing detection thresholds for Survey data of the EMFISIS instrument on Van Allen Probes A and B, respectively, contain 5 noise floor tables and rely on a model of the noise cumulative distribution function for power spectral densities of the magnetic field fluctuations. The validity of this model is demonstrated in Fig. 8, where the experimentally determined values of 1^st^–99^th^ percentiles well correspond to the model. The other 4 noise intervals for Van Allen Probe A, from which the other noise floor tables were constructed, also yield very consistent results, as well as all 5 noise intervals for Van Allen Probe B.

**Fig. 10:**
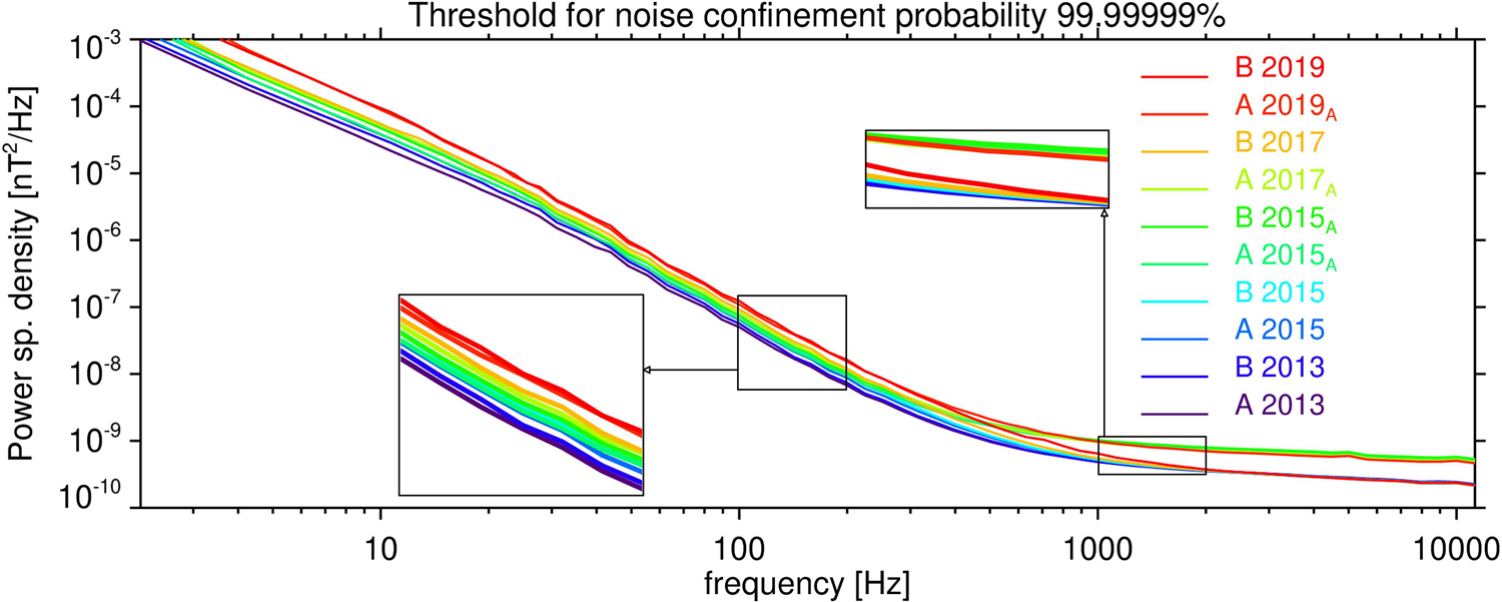
Detection threshold for Survey data of the Van Allen Probes EMFISIS instruments. Detection threshold for *P*_0_ = 10^−7^ as a function of frequency. Two zoom-in inlets show magnified parts of the plot between 100 and 200 Hz and between 1 and 2 kHz, respectively. The noise measurements were obtained from the EMFISIS Waves Survey data from both spacecraft in different years during the Van Allen Probes mission, with both possible settings of the instrument attenuator (index A next to the year in the legend means that the attenuator was on).

It can be expected that the noise floor levels increase during the lifetime of the two spacecraft, as the front-end analog electronics degrade with time. This expectation is validated in Fig. 10, where all 10 noise floors from Data records 1–2 are color coded according to the time during the mission. At lower frequencies below 100 Hz the power-spectral density noise floor slowly increases by a factor 2–4. This effect is negligible at higher frequencies above 1 kHz, where the settings of the instrument play the most important role: the presence of the attenuator increases the noise level by a factor of approximately 2.5.

**Fig. 11:**
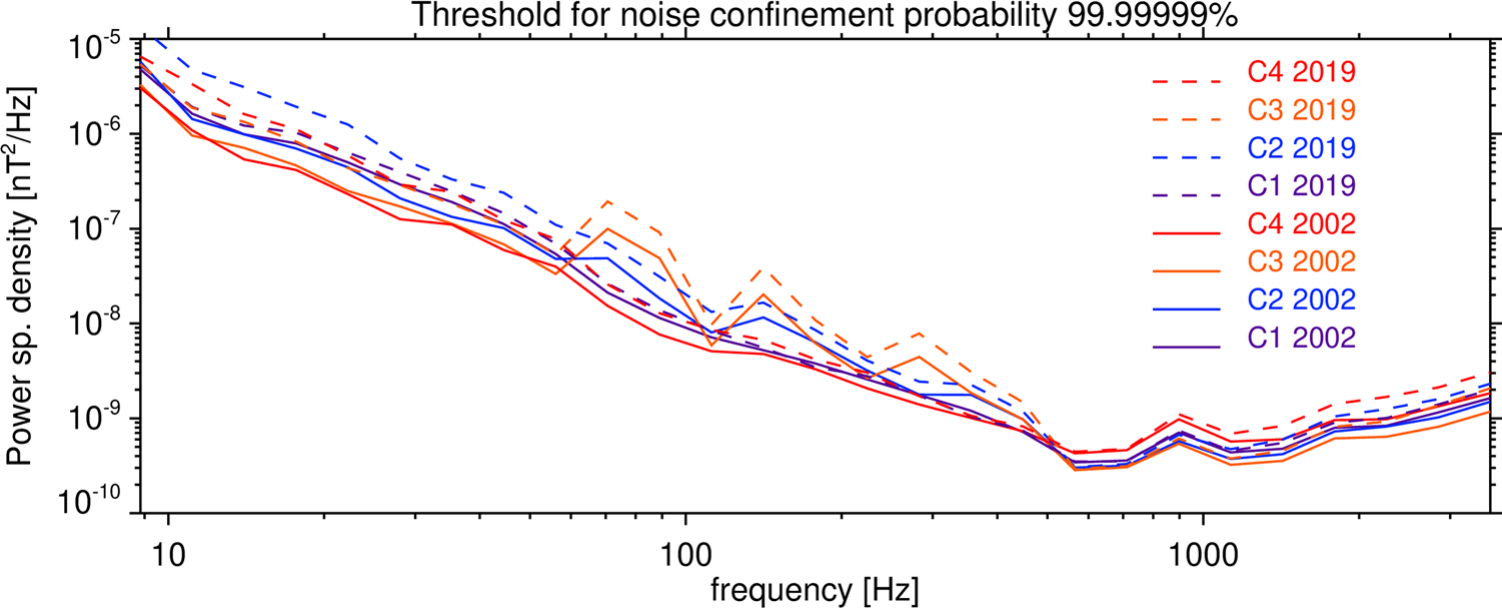
Detection threshold for the Normal mode data of the Cluster 1–4 STAFF-SA instruments. Detection threshold for *P*_0_ = 10^−7^ as a function of frequency obtained from the Normal mode data of the STAFF-SA instrument onboard the four Cluster spacecraft in two different years during the mission, showing substantial differences between the spacecraft, with significant influence of the degradation of the instrument for Cluster 2–4 over 17 years of the mission.

Two tables of detection thresholds for power spectral densities of the magnetic field fluctuations of the Normal mode data of the STAFF-SA instruments are recorded in each of the Data records 3–6 for Cluster 1–4, respectively. These data records rely on another model of the noise cumulative distribution function, whose validity is demonstrated in Fig. 9 for the first noise interval on Cluster 1: the experimentally determined values of 1^st^–99^th^ percentiles again well correspond to this model. Consistent results are also obtained for other noise intervals and other 3 Cluster spacecraft. Figure 11 shows a comparison of all 8 noise floor tables. Increase of the noise floor during the lifetime of the four spacecraft is only very small in this case, typically by a factor of less than 2. The most probable reason is that, unlike Van Allen Probes, Cluster spacecraft didn’t spend much time in the inner radiation belt, and its sensor electronics therefore didn’t substantially degrade with time. Larger differences are observed while comparing the noise floor of different Cluster spacecraft, Cluster 3 being the noisiest one among them at frequencies between 70 and 500 Hz, up to 7 times noisier than Cluster 4.

Data records 7–12 of root-mean-square amplitudes of lower band chorus/exohiss are spatially limited within the magnetospheric region. Its outer limit is set by a magnetopause model^35^, as it is described by the first four paragraphs of the Methods section. Additionally, we limit the Cluster data within a fixed radial distance of 11 Earth’s radii, while the Van Allen Probes measurements are naturally limited by the apogee of the spacecraft orbit at a radial distance of 5.83 Earth’s radii. However, lower band chorus/exohiss emissions usually occur in a more restricted low-density plasmatrough region outside of the plasmapause. To facilitate the selection of relevant measurements we therefore added the 7^th^ data component to data records 7–12, the difference $L-{L}_{{PP}}$ of the equatorial distance of a dipole field line passing through the spacecraft at the time of measurement (with an $L$ value from Eq. 1) from the model plasmapause ${L}_{{PP}}$ ^20^. This model is parametrized by the planetary index Kp of geomagnetic activity, and by the magnetic local time. The difference $L-{L}_{{PP}}$ can be used to define the inner limit of a subset of measurements located in the plasmatrough, considering the uncertainty of the empirical plasmapause model characterized by an RMS error of 0.74 Earth radii^20^. Using the empirical model of the plasmapause might be questionable in the situation when we have measurements of the plasma density onboard the spacecraft (as those shown in Fig. 1a,d) and the plasmapause can be identified from these measurements by sharp density increases. There are, however, three good reasons for which it makes sense to use an empirical model: First, the density measurements are not always available together with the wave measurements or, more importantly, do not show a clear gradient, which would allow us to define the plasmapause position. By selecting only those measurements of lower-band chorus/exohiss, which were close to the sharp density gradients, we would introduce a strong unwanted selection bias into our database of root-mean-square amplitudes of these waves. When using an empirical model, whose results are available for any given time, we do not exclude any wave measurements, even if the model was built on cases with the sharp density gradients.

Second, the plasmapause position from direct measurements is known only at the time when the spacecraft passes through it, while plasmapause position can change between the two spacecraft half-orbits on the time scale of several hours. Linear interpolation of the plasmapause position on these time scales may not be sufficient to capture its motion and to provide us with a reliable estimate of the difference $L-{L}_{{PP}}$. An empirical model of ${L}_{{PP}}$ might be better able to capture sudden changes of geomagnetic activity.

Third, an important practical limitation is that the density measurements use different experimental approaches on different spacecraft. They also have different sampling frequency and measurement precision, different coverage, and different experimental restrictions on the resulting range of density values. Introducing these differences into our database would unnecessarily increase its inhomogeneity compared to using the same empirical model throughout all the data sources.

The particular model that we use in this work was derived from the database of *in situ* observations of over 900 plasmapause crossings by the CRRES spacecraft^20^. It is therefore necessary to validate this model by comparing its output with *in-situ* observations of plasmapause crossings by spacecraft from our dataset. For this validation, we have used the measurements of the local plasma density from the upper hybrid frequency^21^ by the EMFISIS instrument onboard the Van Allen Probes. Similarly to the CRRES model^20^, we have defined a plasmapause crossing as the inner-most passage of the spacecraft through a sharp density change by a factor of at least 5 on a spatial scale lower than one half of the Earth radius along the spacecraft orbit. We identified 13810 such crossings by both Van Allen Probes, it means we use a 15 times larger database than the original CRRES model^16^.

We compared the L values (Eq. 1) of the positions of these crossings with the CRRES model^20^. This model is parametrized by Kp*, which is the running maximum of the Kp index over the time interval between 36 and 2 hours prior to the observation, and by the magnetic local time MLT in hours, converted into the azimuthal angle, $\phi =\frac{\pi }{12}$ MLT. It has a simple functional form with 6 model coefficients (see Table 2), determined by a nonlinear fit to the CRRES plasmapause crossings^20^,

8 $${L}_{{pp}}={{\rm{Kp}}}^{* }{a}_{1}\left[1+{a}_{{mlt}}\,\cos \left(\phi -{a}_{\phi }\right)\right]+{b}_{1}\left[1+{b}_{{mlt}}\,\cos \left(\phi -{b}_{\phi }\right)\right].$$

**Table 2:** Model parameters from Eq. 8, with resulting standard deviations σ for the CRRES plasmapause model^20^ and for a new Van Allen Probes (RBSP) plasmapause model.

Model	*a*_1_	*a*_*mlt*_	(12/π)*a*_ϕ_	*b*_1_	*b*_*mlt*_	(12/π)*b*_ϕ_	σ (R_E_)
**CRRES**	−0.39 ± 0.02	−0.34 ± 0.05	16.6 ± 0.2	5.60 ± 0.10	0.12 ± 0.17	3.0 ± 1.0	0.74
**RBSP**	−0.43 ± 0.01	−0.22 ± 0.02	19.0 ± 0.4	5.97 ± 0.03	0.04 ± 0.01	6.8 ± 0.5	0.75

**Fig. 12:**
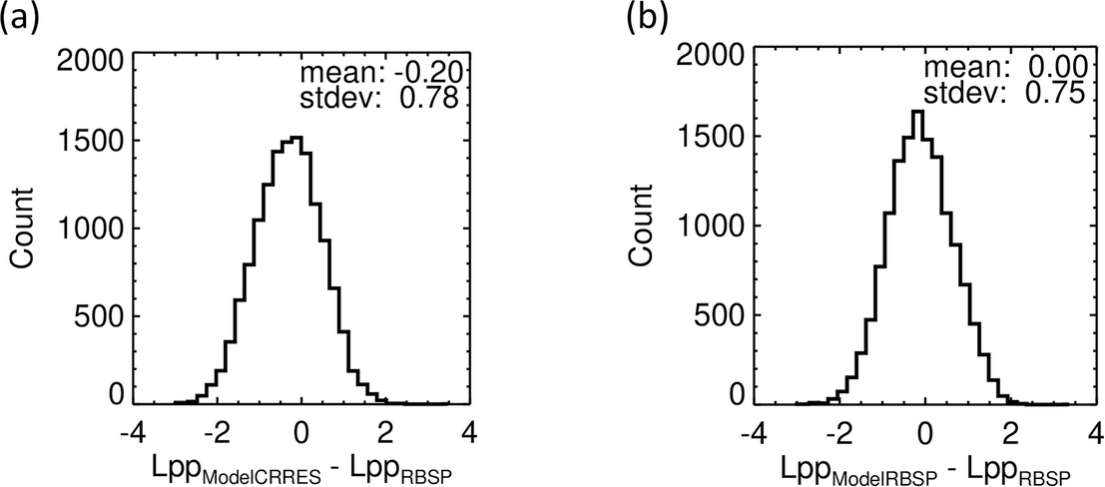
Histograms of differences of the plasmapause models from 13810 plasmapause crossings by both Van Allen Probes. (**a**) Differences of plasmapause L parameter from the CRRES model^20^ and the L parameter from Eq. 1 of the Van Allen Probes (RBSP) plasmapause crossings. (**b**) The same differences but for the updated model based on a nonlinear fit of the Van Allen Probes (RBSP) plasmapause crossings.

Results of the comparison with the Van Allen Probes crossings are shown in Fig. 12a. The differences reveal a small inward bias or 0.2 Earth’s radii of the model, and an approximately Gaussian distribution of the model values around the actual plasmapause position with a standard deviation of 0.78 Earth’s radii. This is very close to the original performance of the model with the CRRES data (reaching a standard deviation σ of 0.74 Earth’s radii, see Table 2).

Results of a nonlinear fit of the six parameters of the same functional form of the model according to Eq. 8 to our large database of 13810 plasmapause crossings by Van Allen Probes are also summarized in Table 2 and in the histogram of differences in Fig. 12b. This new fit leads to a slightly improved performance of the model, removing naturally the bias against the Van Allen Probes crossings, and reaching a standard deviation of 0.75 Earth’s radii.

Although the Data records 7–12 were obtained from different instruments and onboard analysis procedures of the Survey data of the EMFISIS Waves instrument on Van Allen Probes (RBSP) and of the Normal mode data of the STAFF-SA instrument on Cluster spacecraft, considering the detection thresholds improves the homogeneity of contributions from these different data sources to our database. The overall results show similar median and mean values for all the 6 data records of the root-mean-square amplitudes B_w_ of lower-band chorus and exohiss (see Table 3) for overlapping measurements outside of the model plasmapause^20^, at magnetic latitudes within 19.9° from the equator, radial distances below 6.2 Earth’s radii, and in the common time interval between February 13, 2013 and July 16, 2019.

**Table 3:** Parameters of the Data records 7–12 of measurements of lower-band chorus and exohiss.

Data record	Spacecraft	Overlapping measurements		
		Number	Median B_w_ (pT)	Mean B_w_ (pT)
**7**	Van Allen Probe A	1.23 × 10^7^	2.32	7.56
**8**	Van Allen Probe B	1.29 × 10^7^	2.20	7.38
**9**	Cluster 1	8.63 × 10^5^	2.17	7.68
**10**	Cluster 2	8.47 × 10^5^	2.18	7.48
**11**	Cluster 3	4.40 × 10^5^	2.17	7.37
**12**	Cluster 4	4.67 × 10^5^	2.14	7.68

Number of measurements, median, and average B_w_ for measurements overlapping in space and time outside the model plasmapause^20^.

**Fig. 13:**
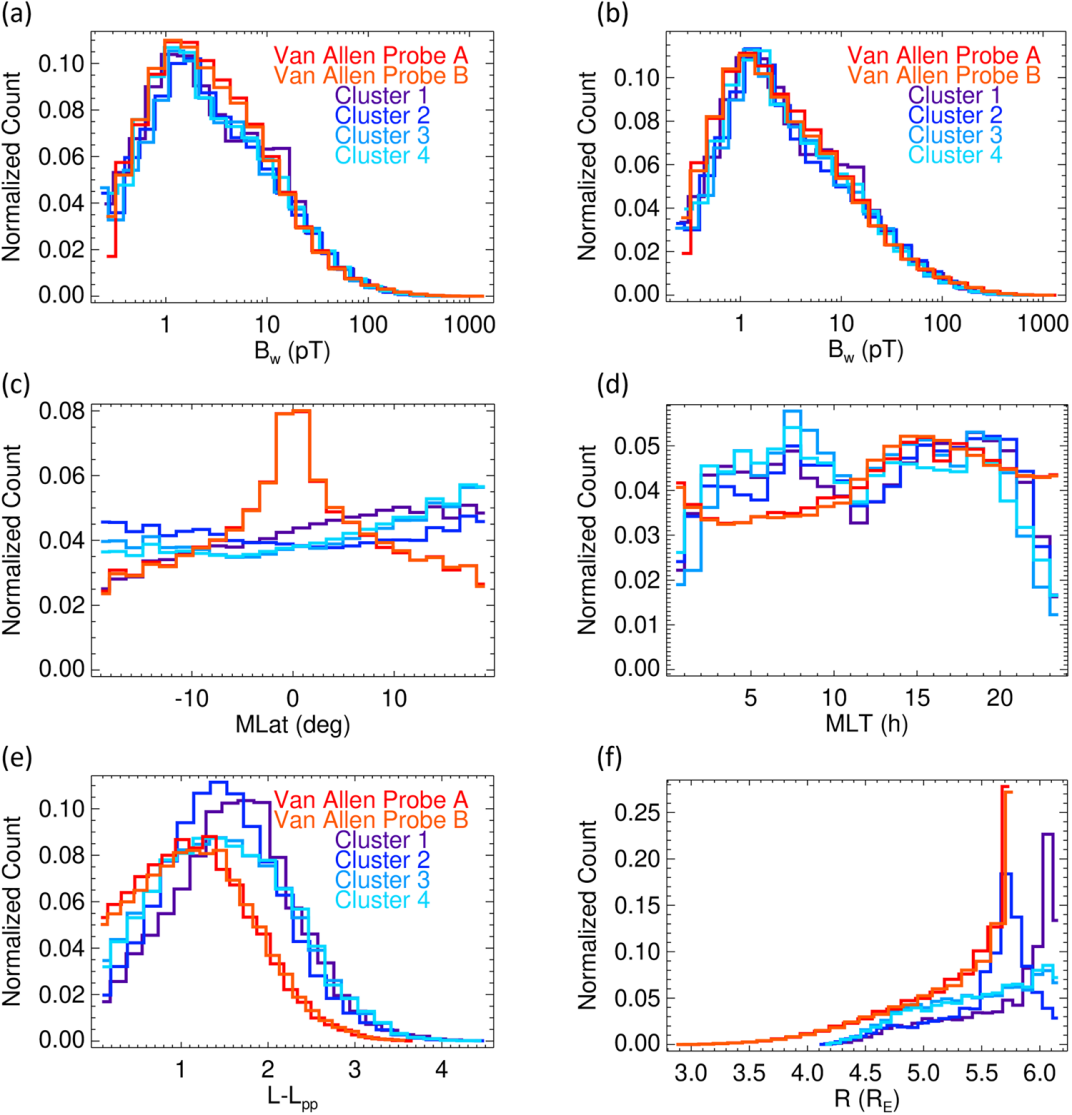
Distributions root-mean-square amplitudes and spatial coverage of overlapping measurements of lower-band chorus and exohiss. Color coded distributions are given for data records from Table 3. (**a**) Distributions of root-mean-square amplitudes B_w_ shown as histograms in 25 logarithmic bins, normalized by the number of overlapping measurements in each data record; (**b**) the same but for the restricted subset of data on the morning side with magnetic local time of 0–12 h; (**c**) distributions of magnetic latitudes for the data records from (**a**); (**d**) distributions of magnetic local time for the data records from (**a**); (**e**) distributions of equatorial distances from the model plasmapause for the data records from (**a**); (**f**) distributions of radial distances for the data records from (**a**).

Figure 13a shows a detailed comparison of the distributions of B_w_ of lower-band chorus and exohiss used in Table 3 for different data sources. As a typical distribution has a heavy tail, being close to the log-normal distribution, we use normalized histograms with logarithmic bins, which show similar distributions for all the data records spanning from a fraction of a picotesla to over 1 nanotesla. Slight differences in the shape of the distributions from Van Allen Probes and from Cluster between 2 and 10 pT (Fig. 13a) are minimized if we select only the measurements on the morning side at magnetic local time of 0–12 h, where we expect predominant occurrence of lower-band chorus and exohiss (Fig. 13b). This effect is partly related to differences in the orbital coverage and to differences of sampling of intense events around the equator (Fig. 13c–f), as it is also detailed in the Usage Notes section.

## Usage Notes

Custom code to handle the tables of detection thresholds (Data records 1–6) is available on 10.6084/m9.figshare.29433593. It performs simple linear interpolation of noise floors in a selected data record.

For example, the Data record 1 for Van Allen Probe A contains 12 time nodes with references to 5 noise floors, numbered from 0 to 4:

2012-01-01T00:00:00.000 0 0

2013-02-25T16:00:00.000 0 0

2015-02-16T11:59:54.262 1 2

2015-09-15T21:29:50.336 2 1

2015-09-15T21:48:20.337 1 2

2015-10-19T20:54:45.267 2 1

2015-10-19T21:12:33.270 1 2

2015-11-07T00:49:01.337 2 1

2015-11-07T16:17:49.242 1 2

2017-01-24T01:30:00.000 3 3

2019-07-30T13:40:00.000 4 4

2020-01-01T00:00:00.000 4 4

If detection thresholds for 2014-09-12T00:00 UT are required, then the noise floors 0 and 1 are linearly interpolated to this time for each frequency channel, between 2013-02-25T16:00:00 and 2015-02-16T11:59:54.262, respectively. If detection thresholds for 2017-02-21T00:00 UT are required, then the noise floors 3 and 4 are similarly interpolated.

A summary of all data records of detection thresholds is given in Table 4. The numbers of time nodes, noise floor tables, and frequency channels are indicated, as well as the times of the beginning of the first and the last noise intervals, which are used to derive the noise floor tables in each data record. The results from the first interval are also used for any time before this first noise interval was captured and, similarly, the noise floor table from the last noise interval is used after the time of its beginning. To avoid unnecessary extrapolation, the times of the first and last noise floor were defined as close as possible to the first and last measurement, respectively, for a given instrument in our database.

**Table 4:** Data records of detection thresholds.

Data record	Spacecraft	First noise floor UT	Last noise floor UT	Time nodes	Noise floors	Freq. channels
1	Van Allen Probe A	2013-02-25T16:00:00	2019-07-30T13:40:00	12	5	65
2	Van Allen Probe B	2013-02-20T16:30:00	2019-07-05T07:30:00	7	5	65
3	Cluster 1	2002-03-08T18:00:00	2019-11-20T07:00:00	4	2	27
4	Cluster 2	2002-02-20T00:00:00	2019-11-02T04:00:00	4	2	27
5	Cluster 3	2002-02-20T00:00:00	2019-11-02T04:00:00	4	2	27
6	Cluster 4	2002-02-20T00:00:00	2019-11-04T10:00:00	4	2	27

Table 5 gives summary information about the data records of root-mean-square amplitudes of lower band chorus/exohiss, including the time coverage from separate data sources, and number of measurements from each of them. The last column gives the number of measurements in which the lower band chorus/exohiss waves were detected. In the separate data records this number is equal to the number of measurements with nonzero root-mean-square amplitudes B_w_ amplitudes, for which the measured right-hand polarized power spectral density overpassed the detection threshold in at least one of the frequency channels contained within the lower band chorus/exohiss frequency band, according to Eq. 8.

**Table 5:** Data records of root-mean-square amplitudes of lower band chorus/exohiss.

Data record	Spacecraft	First record UT	Last record UT	Total Number	B_w_ > 0 Number
7	Van Allen Probe A	2013-02-12T05:00:03	2019-10-14T00:00:00	2.58 × 10^7^	1.65 × 10^7^
8	Van Allen Probe B	2013-02-13T02:00:05	2019-07-16T12:59:59	2.51 × 10^7^	1.65 × 10^7^
9	Cluster 1	2001-01-07T06:32:05	2020-04-30T23:59:52	1.84 × 10^7^	7.28 × 10^6^
10	Cluster 2	2001-01-07T06:29:17	2020-04-30T23:59:51	1.77 × 10^7^	6.81 × 10^6^
11	Cluster 3	2001-01-07T00:48:18	2020-04-30T23:59:50	1.80 × 10^7^	7.61 × 10^6^
12	Cluster 4	2001-01-07T00:50:14	2020-04-30T23:59:49	1.89 × 10^7^	7.31 × 10^6^

**Fig. 14:**
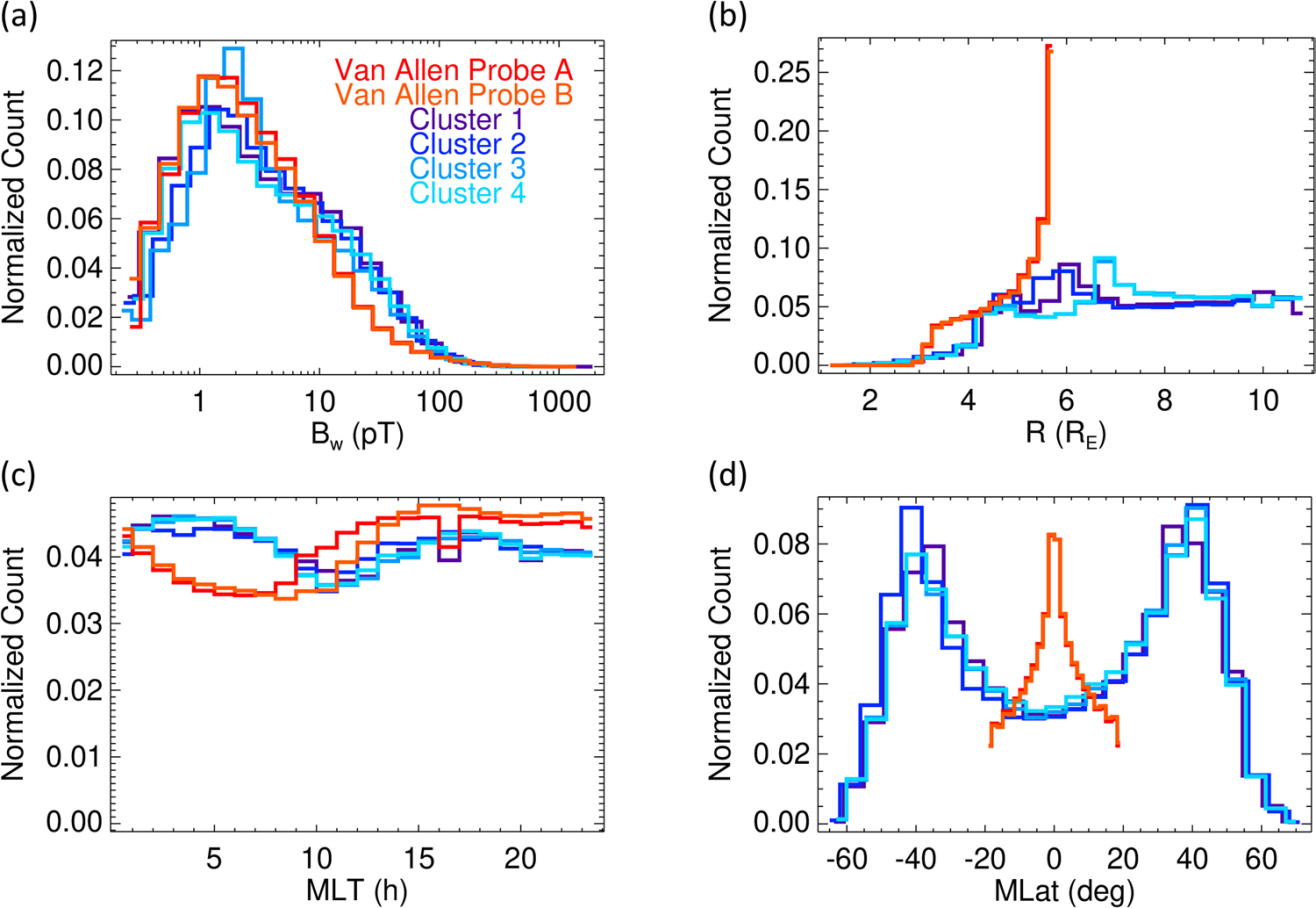
Spatial coverage of the Van Allen Probes and Cluster data records 7–8, and 9–12, respectively. (**a**) Number of measurements in 25 bins of root-mean-square amplitudes B_w_ normalized to the number of measurements above the noise thresholds, shown in Table 5. (**b**) Number of measurements in 25 bins of radial distance R normalized to the total number of measurements shown in Table 5. (**c**) The same but for the magnetic local time MLT. (**d**) The same but for the magnetic latitude MLat.

Data records 7–12 originate from 6 different spacecraft from 2 missions, with different measurements methods, different frequency channels, and different spatial coverage. Although the distributions of root-mean-square amplitudes obtained from all available data are similar (Fig. 14a), and magnetic local time is nearly uniformly sampled (Fig. 14c), the coverage of radial distances (Fig. 14b) and magnetic latitudes (Fig. 14d) is very different for the Cluster and Van Allen Probes missions. Caution must therefore be exercised when joining these data records for a common analysis, taking into account the differences of the analyzed regions and time intervals.

## Data Availability

Analysis in this paper was done using the Interactive Data Language software available from NV5 Geospatial Co. Custom code to handle the tables of detection thresholds (Data records 1–6) is available on 10.6084/m9.figshare.29433593.
